# Large Animal Model of Osteoporotic Defect Healing: An Alternative to Metaphyseal Defect Model

**DOI:** 10.3390/life11030254

**Published:** 2021-03-19

**Authors:** Markus Rupp, Christoph Biehl, Deeksha Malhan, Fathi Hassan, Sameh Attia, Sebastian Rosch, Annemarie B. Schäfer, Erin McMahon, Marian Kampschulte, Christian Heiss, Thaqif El Khassawna

**Affiliations:** 1 Experimental Trauma Surgery, Faculty of Medicine, Justus-Liebig University, Aulweg 128, 35392 Giessen, Germany; markus.rupp@klinik.uni-regensburg.de (M.R.); christoph.biehl@chiru.med.uni-giessen.de (C.B.); deeksha.malhan@charite.de (D.M.); fathihassan@hotmail.com (F.H.); sebastian.rosch@medizin.uni-leipzig.de (S.R.); annemarie.schaefer@med.uni-muenchen.de (A.B.S.); christian.heiss@chiru.med.uni-giessen.de (C.H.); 2 Department of Trauma, Hand and Reconstructive Surgery, Faculty of Medicine, Justus-Liebig University, Rudolf-Buchheim- Str. 7, 35392 Giessen, Germany; 3 Department of Oral and Maxillofacial Surgery, Faculty of Medicine, Justus-Liebig University Giessen, Klinik Str. 33, 35392 Giessen, Germany; sameh.attia@dentist.med.uni-giessen.de; 4 Department of Veterinary Science, University of Kentucky, Lexington, KY 40503, USA; erin.mcmahon@uky.edu; 5 Laboratory of Experimental Radiology, Faculty of Medicine, Justus-Liebig-University Giessen, Schubertstrasse 81, 35392 Giessen, Germany; marian.kampschulte@radiol.med.uni-giessen.de

**Keywords:** osteoporosis, bone healing, sheep model, 3R

## Abstract

Osteoporosis is a common metabolic disorder diagnosed by lower bone density and higher risk of fracture. Fragility fractures because of osteoporosis are associated with high mortality rate. Deep understanding of fracture healing in osteoporosis is important for successful treatment. Therefore, the FDA approved the use of small and large animal models for preclinical testing. This study investigated the clinical relevance of a fracture defect model in the iliac crest of the osteoporotic sheep model and its several advantages over other models. The osteoporosis was achieved using ovariectomy (OVX) in combination with diet deficiency (OVXD) and steroid administration (OVXDS). Fluorochrome was injected to examine the rate of bone remodelling and bone mineralization. The defect areas were collected and embedded in paraffin and polymethyl metha acrylate (PMMA) for histological staining. OVXDS showed significantly lower bone mineral density (BMD) and bone mineral content (BMC) at all time points. Furthermore, variations in healing patterns were noticed, while the control, OVX and OVXD showed complete healing after 8 months. Bone quality was affected mostly in the OVXDS group showing irregular trabecular network, lower cortical bone thickness and higher cartilaginous tissue at 8 months. The mineral deposition rate showed a declining pattern in the control, OVX, and OVXD from 5 months to 8 months. One the contrary, the OVXDS group showed an incremental pattern from 5 months to 8 months. The defect zone in osteoporotic animals showed impaired healing and the control showed complete healing after 8 months. This unique established model serves as a dual-purpose model and has several advantages: no intraoperative and postoperative complications, no need for fixation methods for biomaterial testing, and reduction in animal numbers, which comply with 3R principles by using the same animal at two different time points.

## 1. Introduction

Osteoporosis is a systemic skeletal disease characterized by lower bone mass and declined bone quality resulting in an increased risk of fractures [1,2]. Osteoporosis causes more than 8.9 million fractures each year, resulting in an osteoporotic fracture every 3 s worldwide [3]. Identifying patients who are at higher fracture risk for successful treatment of osteoporotic fracture is challenging for orthopedic surgeons. Further, the underlying mechanism behind the pathogenesis of osteoporosis remain unknown. Therefore, preclinical research is carried out to understand the underlying molecular mechanism and to find suitable therapeutics to treat osteoporotic fractures.

The preclinical testing of different biomaterials and tissue engineering approaches are mostly carried out using in vitro systems [4,5,6,7,8] and small animal models [9,10,11,12,13]. However, these models present several limitations such as incompatibility with the human condition and transferability of information. On the contrary, large animal models, especially sheep, have proven more helpful in translational research with respect to therapeutics testing [14,15,16].

Sheep have several advantages like body weight, long bones, and comparable macrostructure to be a suitable animal model for the study of osteoporosis and defect healing. Osteoporotic fractures occur mostly in the metaphyseal bone region but the healing mechanism is not yet known. Previous studies reported the application of biomaterials in sheep defect healing model to evaluate cellular and structural changes [17,18,19]. However, the studies were limited to a specific time point throughout the healing process because of increased complications with this animal model. Further, the osteoporotic bone status was not yet established in the animal model. Therefore, a clinically relevant defect healing model in an osteoporotic animal remains unknown.

In this study, we hypothesized that the defect zone region in the iliac crest of the osteoporotic sheep can serve as a preclinical model for biomaterial testing. Therefore, we used the previously established sheep osteoporotic model [20] achieved by ovariectomy, diet deficiency, and steroid administration for 8 months. Radiological and histological techniques were applied on the defect zone. The study aims to examine the healing progression in the osteoporotic group compared with the control in the defect zone.

The data also confer the advantage of the iliac crest defect region to understand osteoporotic fracture healing over the metaphyseal bone defects. More importantly, the use of the same animal model for two different studies at two different time points lessens the use of experimental animals and results in less stress for the animals. This study complies with the 3R (reduce, replace, refine) principles of the animal welfare society [21].

## 2. Materials and Methods

### 2.1. Experimental Design

Female merino land sheep (n = 31, average age = 5.5 years) were used to investigate the defect zone healing. Animals were randomly divided into: (1) nonoperated control group (control, n = 8); (2) bilaterally ovariectomized group (OVX, n = 7); (3) bilaterally ovariectomized and deficient diet treatment (OVXD, n = 8); and (4) triple treatment group; bilaterally ovariectomized, deficient diet and intramuscular steroid-suspension treatment (OVXDS, n = 8).

**Figure 1:**
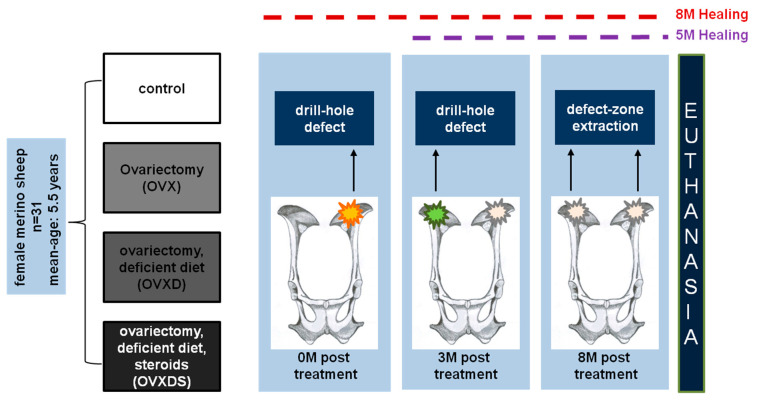
Pictorial representation of the study design using sheep osteoporotic model.

Two weeks after recovery from bilateral ovariectomy, the deficient diet was given to OVXD and OVXDS groups. Iliac crest biopsies were taken and bone defects were created using diamond trepan of 7.5 mm diameter and 25 mm long at (M = Month) 0 M from the left side and at 3 M after treatment from the right side which reduced the animal number by the half. The defect diameter was chosen based on the clinical recommendation of trauma surgeons of the department as the largest possible cylindrical defect without disturbing the bicortical walls of the iliac crest. The length of the defect recapitulated a critical size defect in osteoporosis as previously described [22,23,24]. All groups were then examined 8 M post-treatment resulting in a healing time of 8 M for the 0 M defect zone and 5 M for the 3 M defect zone. To animal welfare concerns, premedication of 10 mg/kg ketamine hydrochloride (100 mg/mL Ketavet, Bela-Pharm GmbH and Co KG, Vechta, Germany), 0.01 mL/kg xylazine (2% Rompun, Bayer AG, Berlin, Germany), 0.3 mg/kg midazolam (5 mg/mL Midazolam Rotexmedica, Rotexmedica GmbH, Trittau, Germany), and 0.01 mg/kg atropine (0.5 mg/mL Atropinsulfat, Braun Melsungen AG, Melsungen, Germany) were given to the animals prior to the ovariectomy. Subsequently, anaesthesia was administered using 2 mg/kg propofol (20 mg/mL propofol, Fresenius Kabi, Bad Homburg, Germany) and 2 µg/kg fentanyl (50 µg/mL Fentanyl-Hameln, Hameln Pharmaceutical GmbH, Hameln, Germany). Pain killers were given to the animals before and after surgery for up to 5 days using 0.01 mg/kg buprenorphine hydrochloride (0.3 mg Temgesic ampoules, RB Pharmaceuticals GmbH, Heidelberg, Germany) and 0.5 mg/kg meloxicam (20 mg/mL Metacam, Boehringer Ingelheim Vetmedica GmbH, Ingelheim am Rhein, Germany). Animals were daily monitored after surgery, until no antibiotics or painkillers were administered. Animals were euthanized after 8 M of treatment by intravenous administration of 50 mg/kg pentobarbital (Anestesal, Pfizer, Deutschland GmbH, Berlin, Germany) under anaesthesia. After euthanasia, defect zones were collected (Figure 1) for analysis.

### 2.2. Animal Diet

Control and OVX groups received a standard diet (S6189-S010; Sondermischung Schaf, 4 mm pellet, ssniff-Spezialdiäten GmbH, Soest, Germany) whereas the OVXD and OVXDS groups received a diet deficient in calcium and vitamin D2/3. Both the standard and the deficient diets were offered twice a day along with ad libitum hay.

### 2.3. Steroid Administration

Two weeks postovariectomy, sheep of the OVXDS group received a biweekly dosage of 320 mg methylprednisolon/sheep as previously described [21,25,26] (Depot- Medrate^®^ ad us. vet 40 mg/mL injection suspension, Pfizer Deutschland GmbH, Berlin, Germany).

### 2.4. Dual Energy X-ray Absorptiometry (DXA)

Alterations in bone mineral density (BMD) and bone mineral content (BMC) were investigated in the pelvis region throughout the progression of osteoporosis (0 M, 3 M and 8 M). Lunar prodigy software (version 13.40, GE Healthcare, Darmstadt, Germany) was used to carry out the scans. Device calibration was carried out prior to scans according to the manufacturer’s protocol. The sheep was anaesthetized and placed under intubation in prone position with splayed limbs. The neck and head were adjusted and fixated using the attenuation free accessories. A whole-body scan was carried out by DXA (lunar prodigy, GE Healthcare, Munich, Germany).

### 2.5. Micro-Computed Tomography (µCT)

µCT serves as a precise and clinically tested method to assess bone geometry and structural changes [25,26].

Cylindrical samples from the iliac crest were imaged using the µ-CT system SkyScan 1173 (Bruker MicroCT, Kontich, Belgium). Scanning parameters were set as follows: tube voltage: 45 kVp, tube current: 175 µA, rotation step width: 0.3°, projections: 800, frame averaging: 4-fold. For beam filtration a 0.5 mm aluminum filter was used. NRecon-Software (Bruker microCT, Kontich, Belgium) with a Gaussian filter (smoothing kernel = 2) was utilized for reconstruction of isotropic voxels: 7.1 µm side length.

### 2.6. Fluorochrome Labelling

Three animals from each group were randomly chosen for in vivo fluorochrome labelling to examine the rate of bone remodelling and bone mineralization. Fluorochromes were purchased from Sigma-Aldrich (Munich, Germany) and administered after pH adjustment and filter sterilization. Calcein green (C0875) was injected 14 days and 7 days prior to euthanasia while alizarin red (A3882) was injected 15 days prior to euthanasia. After euthanasia, samples were collected and embedded in polymethylmethacrylate PMMA (Technovit 9100, Heraeus Kulzer, Hanau, Germany) according to standardized protocol (22) and then sectioned and ground into 10–20 µm thick slices.

### 2.7. Sample Preparation and Histology

Both decalcified and undecalcified histological analyses were conducted on the defect zone samples to investigate cellular and structural changes underlying healing. After euthanasia, defect zones were collected and freed from soft tissue. The samples were fixed in 4% paraformaldehyde (PFA) and then decalcified using 4% PFA and 14% ethylenediaminetetraacetic acid (EDTA) for 8 weeks at 4 °C. After embedding, paraffin blocks were sectioned into 5 µm thick slices using motorized rotary microtome (Thermo/Microm HM355S, Thermo Scientific GmbH, Karlsruhe, Germany), whereas the fluorochrome labelled samples were embedded in PMMA medium.

Decalcified sections were used for toluidine blue staining [27,28,29] to carry out the qualitative analysis of the defect zone across time points.

### 2.8. Image Capturing and Quantitative Evaluation

Toluidine blue stained sections were imaged using a Axioplan 2 photographic microscope equipped with photo module Axiphot2 (Carl Zeiss, Wetzlar, Germany). Fluorochrome labelled slices were imaged using a Leica microscope (Leica DM5500 photomicroscope equipped with a DFC7000 camera and LASX software version 3.0; Leica Microsystem Ltd., Wetzlar, Germany). Fluorescence illumination system (X-Cite^®^200DC, Lumen Dynamics Group Inc., Mississauga, ON, Canada) from Leica microscope was used to capture images of fluorochromes.

### 2.9. Ethical Statement

This study is a part of a transregional project aimed at understanding the cellular and structural changes underlying osteoporosis using a large animal model. This study was conducted in compliance with our institutional regulation and German animal protection laws. The experimental study was approved by the ethical committee of the local governmental institution (Regierungspäsidium Darmstadt, permit number/application number: F31/36, Reference number: V54—19c 20/15).

### 2.10. Statistical Analysis

A descriptive statistical analysis was conducted to test the normal distribution of data. Data were analysed using Statistical Package PASW 20.0 (IBM, SPSS Inc., Armonk, NY, USA). Additionally, the Kolmogorov–Smirnov test was used to test normality of data. Statistical significance test for nonparametric distribution was carried out using Mann–Whitney U test. Bar graphs were presented as mean ± standard of error (SEM).

## 3. Results

### 3.1. In Vivo DXA Measurements Showed Bone Loss in OVXDS at 8 M Post-Treatment

**Figure 2:**
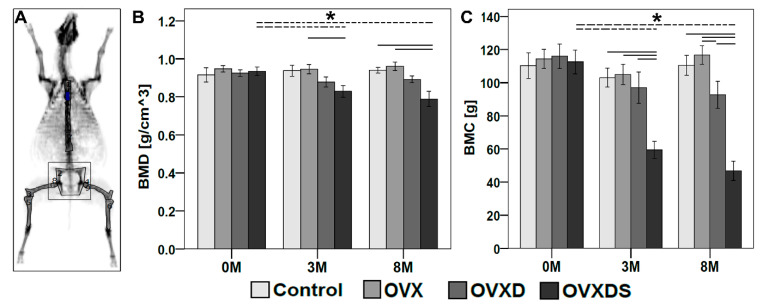
Radiological testing using Dual Energy X-ray Absorptiometry (DXA) revealed deteriorated bone density in the group with triple treatment (OVXDS) after 8 M. In vivo DXA was carried out at 0 M, 3 M, and 8 M to monitor the establishment of osteoporotic bone status in the pelvis region. (**A**) Sheep whole body scan and the selection of region of interest to evaluate the bone status at individual regions. (**B**) OVXDS showed significantly lower bone mineral density (BMD) at 8 M compared to 0 M and 3 M. BMD was lower in OVXDS at all time points compared with other groups. (**C**) OVXDS showed significantly lower BMC at 8 M compared to 0 M and 3 M. BMC was lower at all time points compared with other groups. (n = 8/time point/group (Control, OVXD, OVXDS); n = 7/time point/group (OVX); * = *p* ≤ 0.05: Mann–Whitney U test).

DXA is used as a gold standard to diagnose bone loss among osteoporotic patients. Therefore, the establishment of osteoporotic bone status in the sheep model was monitored before and after treatment using in vivo DXA. BMD and BMC were measured from the pelvis region at 0 M, 3 M and 8 M (Figure 2). Control and OVX groups showed no change in BMD during different time points, while, as expected, OVXD showed lower BMD at 8 M when compared to 0 M and 3 M (Figure 2B). OVXDS showed significantly higher BMD at 0 M when compared to 3 M (*p* = 0.021) and 8 M (*p* = 0.015). All four groups showed no significant differences in BMD at 0 M (Figure 2B). However, OVXDS showed lower BMD at 3 M when compared with the control, OVX, and OVXD. BMD was significantly lower in OVXDS at 3 M when compared with OVX (*p* = 0.014). At 8 M, OVX showed higher BMD when compared with other groups, while OVXDS showed lower BMD when compared with other groups (Figure 2B). OVXDS showed significantly lower BMD when compared with Control (*p* = 0.015) and OVX (*p* = 0.0059). No other significant differences were observed. All four groups showed lower BMC at 3 M when compared to 0 M (Figure 2C), while Control and OVX showed higher BMC after 8 M when compared to 3 MOVXD and OVXDS showed lower BMC after 8 M when compared to 0 M and 3 M (Figure 2C). BMC in OVX and OVXD groups was higher at 0 M when compared with Control and OVXDS. OVXDS showed significantly lower BMC at 3 M (*p* = 0.00031) and 8 M (*p* = 0.00015) when compared to 0 M. BMC was higher in OVX and lower in OVXDS at 3M compared with other groups. OVXDS showed significantly lower BMC at 3M when compared with Control (*p* = 0.001), OVX (*p* = 0.014), and OVXD (*p* = 0.015). At 8 M, BMC was higher in OVX when compared with other groups (Figure 2C) whereas, OVXDS showed lower BMC when compared with other groups at 8 M. OVXDS showed significantly lower BMC at 8 M when compared with Control (*p* = 0.00015), OVX (*p* = 0.00031), and OVXD (*p* = 0.00031). BMC was significantly lower in OVXD when compared with OVX (*p* = 0.029). No other significant differences were observed. The significant drop in both BMD and BMC after 8 M in OVXDS group affects the bone-microarchitecture within the defect zone. Therefore, ex vivo µCT is required to evaluate the healing in the OVXDS defect zone and bone quality.

### 3.2. Radiological Testing Using µCT Showed Impaired Healing of Defect Zone in OVXDS after 8 M

**Figure 3:**
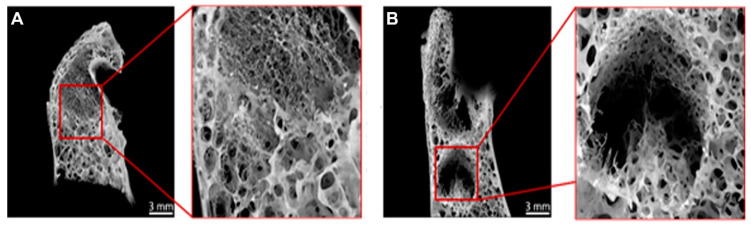
Ex vivo µCT scans from OVXDS samples showed complete healing and impaired healing. µCT scans were carried out to examine whether the defect zone healed completely in OVXDS after 8 M. (**A**) Healed defect zone in the OVXDS group was seen. (**B**) Sample from OVXDS showed impaired healing of defect zone.

The deteriorated bone density in the OVXDS group after 8 M encouraged evaluation of the progress of defect healing. Therefore, ex vivo µCT scans were carried out (Figure 3).

The Control, OVX, and OVXD groups showed complete healing after 8 M of treatment, whereas the OVXDS group showed variation in the healing pattern. One out of eight OVXDS samples showed the complete healing of the defect zone (Figure 3A), while the rest showed impaired healing of the defect zone after 8 M (Figure 3B). Besides impaired healing, OVXDS also showed trabecular loss after 8 M. Therefore, a descriptive analysis of trabecular and cortical bone quality in the defect zone across time points is required.

### 3.3. Impaired Healing in OVXDS Defect Zone Reflects Trabecular Thinning and Failed Fractured Callus Mineralization

**Figure 4:**
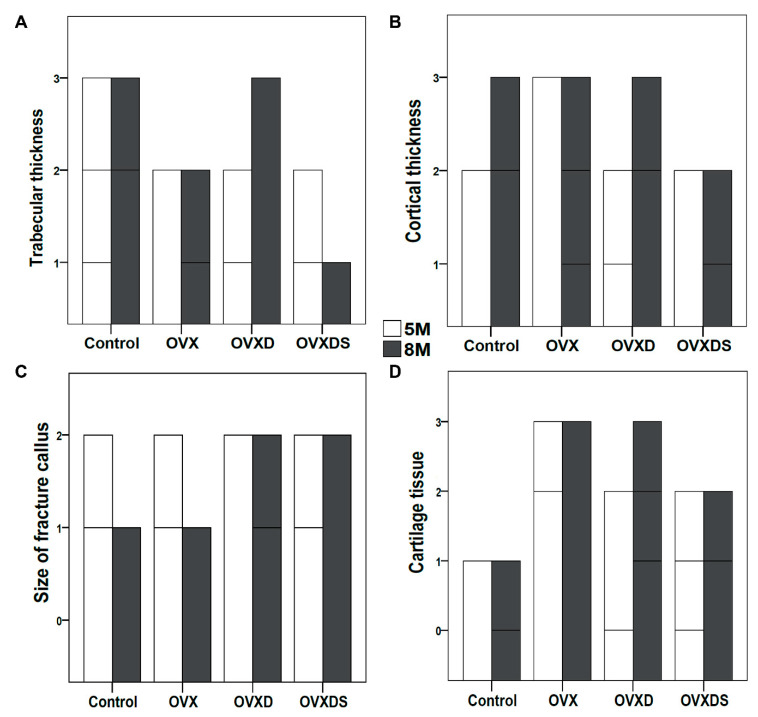
Frequency analysis of bone quality showed deteriorated thickness and delayed healing in OVXDS after 8 M. Toluidine blue stained sections from defect zone after 5 M and 8 M of healing were evaluated to grade the bone quality. (**A**) OVXDS showed lower trabecular thickness after 8 M. (**B**) Cortical thickness was lower in OVXDS at both 5 M and 8 M. (**C**) The size of fractured callus remains unchanged in OVXDS after 8 M. (**D**) Higher amount of cartilage tissue was seen in OVX, OVXD, and OVXDS (n = 8/time point/group (Control, OVXD, OVXDS); n = 7/time point/group (OVX)).

The integrity of trabecular bone and cortical bone and fractured callus mineralization is essential for the complete healing of the defect zone. To assess the defect zone and the quality of the bone, toluidine blue stain was used. The frequency analysis was carried out to examine the progress at 5 M and 8 M after treatment. The specific focuses of bone features were trabecular thickness, cortical thickness, the size of the fractured callus, and the amount of cartilage tissue (Figure 4). Trabecular thickness is an important factor of bone quality. The control group showed well-connected trabecular mesh at 5 M and 8 M (Figure 4A). The trabecular thickness in the control group was higher at 8 M than at 5 M, whereas OVX showed no change in trabecular thickness at 8 M and 5 M (Figure 4A). On the contrary to OVX, OVXD showed higher trabecular thickness at 8 M when compared to 5 M. As expected, trabecular thinning and an irregular trabecular network were seen in the OVXDS group at 8 M compared to 5 M (Figure 4A). Cortical bone thickness is a necessary parameter to evaluate bone quality. Cortical bone thickness was higher in the Control and OVXD groups at 8 M (Figure 4B). No change in cortical bone thickness was seen in OVX and OVXDS at 8 M compared to 5 M. Overall, cortical thickness was lower in OVXDS when compared with other groups (Figure 4B). The mineralization of the fractured callus is important for healthy bone formation. Interestingly, both the Control and OVX groups showed smaller calluses at 8 M when compared to 5 M (Figure 4C). No change in fractured calluses was seen in OVXD and OVXDS at 8 M and 5 M and it was similar to control and OVX at 5 M (Figure 4C). Cartilaginous tissue mineralization takes place with the progression of healing. Control showed higher amount of cartilage tissue at 5 M, whereas only small patches were seen at 8 M (Figure 4D). OVX showed no change in cartilaginous tissue at 8 M and 5 M. The amount of cartilaginous tissue in both the OVXD and OVXDS groups was higher at 8 M compared to 5 M (Figure 4D). The lower trabecular and cortical bone thickness, along with impaired fractured callus mineralization, points towards an imbalanced bone remodelling rate. Therefore, the investigation of the mineralization rate is required.

### 3.4. Deteriorated Bone Matrix Mineralization Seen in OVXDS Defect Zone after 8 M of Treatment

**Figure 5:**
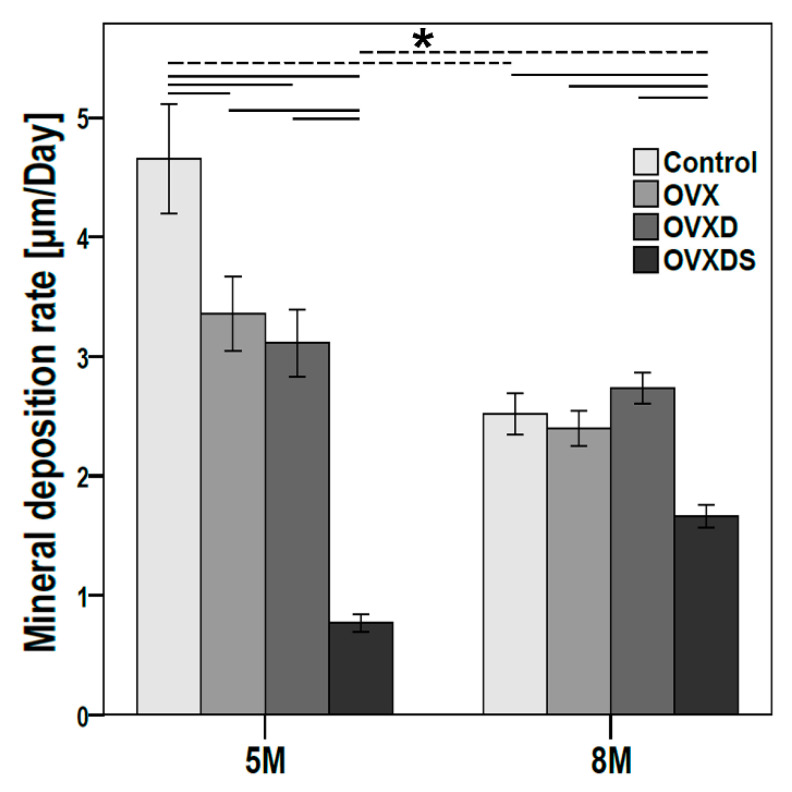
Impaired bone formation rate seen in the OVXDS group after 8 M of healing. Fluorochrome labeling was carried out prior to euthanasia to investigate the changes in bone formation rate (7, 14, and 15 days prior to euthanasia). OVXDS showed significantly higher bone formation rate at 8 M compared to 5 M. The rate of bone formation was lower in OVXDS at both 5 M and 8 M when compared with other groups. (n = 4/time point/group); * = *p* ≤ 0.05: Mann–Whitney U test).

The balance between bone formation and bone resorption is important for bone homeostasis. An imbalanced bone formation rate can result in bone loss. Therefore, fluorochrome labelling was used to examine new bone formation rate at two time points (Figure 5). Control, OVX, and OVXD showed lower mineral deposition rate at 8 M when compared to 5 M (Figure 5), while OVXDS showed higher mineral deposition rate at 8 M than 5 M. Control showed significantly lower mineral deposition rate at 8 M (*p* = 0.000005), while OVXDS showed significantly higher mineral deposition rate at 8 M (*p* = 0.000011). The mineral deposition rate at 5 M was the highest in Control and the lowest in OVXDS when compared with the other groups. Control showed significantly higher mineral deposition rate at 5 M when compared with OVX (*p* = 0.050), OVXD (*p* = 0.042), and OVXDS (*p* ≤ 0.00001). Further, OVXDS showed significantly lower mineral deposition rate at 5 M compared with OVX (*p* ≤ 0.00001) and OVXD (*p* = 0.0006). OVXD showed higher mineral deposition rate compared with the other groups at 8 M (Figure 5), whereas the OVXDS showed lowest mineral deposition rate compared with the other groups at 8 M. The mineral deposition rate in OVXDS was significantly lower at 8 M compared with Control (*p* = 0.0018), OVX (*p* = 0.00072), and OVXD (*p* = 0.00066). The mineral deposition rate showed a similar declining pattern in Control, OVX, and OVXD from 5 M to 8 M, whereas the OVXDS group showed an increment pattern from 5 M to 8 M.

Furthermore, osteoclast mediated bone resorption showed no statistically significant changes between the time points in the experimental groups. However, the control group showed significantly lower number of osteoclasts per square millimeter between 5 M and 8 M (TP, Maximum: Minimum ± SD_*p* value) (5 M, 4: 2 ± 0.4; 8 M, 2:0 ± 0.8 _ *p* ≤ 0.05).

## 4. Discussion

In clinical practice, managing osteoporotic fractures remain challenging. Several studies reported the complexity of osteoporotic fractures and the application of different therapeutics [30,31,32,33]. However, successful treatment options remain unknown despite the use of preclinical animal models.

Most of the studies have used a small animal model to understand the severity of osteoporotic fractures in the metaphyseal region [34,35,36]. However, the major drawback of the small animal model is its incomparability with patients in the clinic. Although the concepts of 3R recommend replacing one species with another that suffers less pain, using small animal models has its limitations. Small animals including mice, rats and rabbits have a small skeletal size and a short life expectancy. Therefore, in bone research this small size can lead to the use of larger numbers of animals for shorter time periods. A major drawback, however, is the different bone microstructure and cellular composition when compared to human bone, which renders small animals less appropriate to test biomaterials. [15,37,38,39]. Many of these limitations are circumvented in large animal models, and for orthopedic research, sheep in particular [15,24,39,40]. Sheep studies showed size similarities between the sheep model and human bone, beside microstructure, metabolism rate, and biomechanical competence [40,41,42]. Therefore, the large animal model is essential for osteoporotic research. However, the increased use of the large animal model for testing potential therapeutics presents ethical, scientific, and economical concerns.

Many studies reported radiological, functional and histological deterioration of bone quality corresponding to osteoporosis induction in sheep. These studies confirmed the sheep validity as a clinically relevant model of osteoporosis [36,41,42]. The model used in this study is considered an acceptable model to recapitulate the clinical effects of osteoporosis on bone metabolism in sheep [20].

According to the 3R principle, a standardized approach is needed to reduce the number of experimental animals and to maintain experimental reproducibility [43]. The model described here was established to study the cellular changes underlying osteoporosis [20] as well as the defect healing mechanism. This study intends to provide researchers with an alternative scientific approach to examine biomaterials in the large animal model at different time points throughout the healing process.

In bone research, the precondition to test biomaterials for osteoporotic fracture healing is described by Schmitz and Hollinger as critical size defect (CSD). The CSD is defined as the smallest intraosseous wound which will not heal by bone remodeling during the lifetime of animal [44,45]. The understanding of fracture repair in sheep models of osteoporosis focused on the femora [22,23,46]** and the spine [47].

The presented study design aims to achieve a clinically relevant recapitulation of the healing process in osteoporotic patients. Clinically, the nonconsolidation of a bone defect after 3–6 months of healing is considered a delayed healing. The lack of full consolidation and remodeling after more than six months postfracture is considered nonunion or pseudarthrosis [48]. The 5 M and 8 M healing periods were designed to differentiate between normal, delayed and nonunion healing. Furthermore, clinical reports showed the mortality of osteoporosis-associated fractures is greatest in the first six months [49].

Therefore, a detailed understanding of fracture healing in the systemically diseased bone may help to decrease mortality and reduce the incidence of pseudarthrosis formation (2–10%) [50].

Despite experimental evidence that bone healing under osteoporotic condition is impaired [51], the longitudinal study of fracture healing in a large animal model with a detailed follow-up of 6 months is not possible. The longest follow-ups with segmental diaphyseal bone defects in young and aged sheep were three and four months, respectively [52,53]. The longest follow-ups result in severe pain for the animals and lower survival rate. However, the model described in this study was used for 8 months and animals showed no added pain or postoperative stress, which was of great advantage.

Because the iliac crest is not exposed to weight bearing regions, its use to study bone defect healing is suitable in a preclinical setup. Besides, the comparable trabecular and cortical bone in the iliac crest to the metaphyseal region makes it a perfect location to test therapeutics. Previously, iliac crest drill-hole defects in goats and sheep were used as metaphyseal healing models [54,55]. Complications of limb drill hole defect models in sheep are reported as being less frequent and tolerable [56].

The application of the iliac crest region to the study of fracture healing brings further concerns, like the impact of mechanical loading on bone repair [57]. However, depending on the location of the fracture, no weight-bearing or partial weight bearing is permitted during the early phase of healing [58]. Further, the conditions of iliac crest bone defects are even more reproducible with weight bearing, as long as the loading of the injured extremities is pain adapted in long bone defects in animal models. The differences of bone healing in early, delayed and steadily increasing weight-bearing, as described by Augat et al., can lead to a bias, which is undesirable in experimental settings. This will not be expected in iliac crest bone defects [59].

The data of this study showed clear characteristics of osteoporosis, like trabecular thinning, lower BMD and BMC in the osteoporotic group after 8 M. Further, the disturbance in the rate of bone formation in OVXDS and large amount of cartilage points towards delayed healing even after 8 M. Thormann et al. (2014) also showed the prevalence of cartilage residue in the metaphyseal defect healing model of osteoporosis [51]. The study findings correlate with the metaphyseal defect model. The established model can be used as dual-purpose model and has several advantages: no intraoperative and postoperative complications, no need for fixation methods for biomaterial testing, two time points, and a reduction in animal numbers.

## 5. Conclusions

The model described in this study presents a low-risk, high-gain critical size defect in the sheep osteoporotic model. We believe the presented model could be helpful for in vivo evaluation of biomaterials for osteoporosis treatment. The use of the iliac crest as a localization of bone defects enables examining several defects in one animal at the same time. Such design results in, standardized bone defects and less painful operations for the animals, which enabled the use of the same animal for the second operation and time point and shorter experimental duration for the used laboratory animals when performed soon after osteoporosis induction. Thusly, this experimental approach completely matches with the 3R principles described by Russell and Burch. To characterize and understand the disturbed bone healing resulting in nonunion in osteoporotic bone defects in the described model, further research is still necessary.

## Data Availability

Data are available upon contact with the corresponding author.
